# Long-term sentinel surveillance of enteroviruses in Gwangju, South Korea, 2011–2020

**DOI:** 10.1038/s41598-023-29461-8

**Published:** 2023-02-16

**Authors:** Min Ji Kim, Ji-eun Lee, Kwang gon Kim, Duck Woong Park, Sun Ju Cho, Tae sun Kim, Hye-young Kee, Sun-Hee Kim, Hye jung Park, Mi Hee Seo, Jae Keun Chung, Jin-jong Seo

**Affiliations:** Department of Infectious Disease Research, Health & Environment Research Institute of Gwangju, 584 Mujin-Daero, Seogu, Gwangju, Republic of Korea 61954

**Keywords:** Molecular biology, Diseases, Molecular medicine

## Abstract

Human enteroviruses (EVs) are associated with a broad spectrum of diseases. To understand EV epidemiology, we present longitudinal data reflecting changing EV prevalence patterns in South Korea. We collected 7160 specimens from patients with suspected EV infections in ten hospitals in Gwangju, Korea during 2011–2020. RNA extraction and real-time reverse transcription polymerase chain reaction using EV-specific probes and primers were performed. EV genotyping and phylogenetic analysis were performed; EVs were detected in 3076 samples (43.0%), and the annual EV detection rate varied. EV infection rates did not differ with sex, and children aged ≤ 4 years were the most prone to EV infection; this trend did not change over time. Overall, 35 different EV types belonging to four distinctive species and rhinoviruses were identified. Although serotype distribution changed annually, the most frequently observed EVs were EV-A71 (13.1% of the cases), CVA6 (8.3%), CVB5 (7.6%), CVA16 (7.6%), CVA10 (7.5%), E18 (7.5%), E30 (7.0%), and E11 (5.0%) during 2011–2020. The predominant EV genotypes by clinical manifestation were CVB5 for aseptic meningitis; EV-A71 for hand, foot, and mouth disease cases; and CVA10 for herpangina. These results will aid the development of vaccines against EV infection and allow comprehensive disease control.

## Introduction

Human enteroviruses (EVs) belong to the genus *Enterovirus* of the Picornaviridae family and are classified into four species: EV A–D. Currently, there are 116 serotypes, including EV-A (25 types), EV-B (63 types), EV-C (23 types), and EV-D (five types). These serotypes are determined by the antigenicity of viral protein 1 (VP1) among other surface proteins, and the genotype is determined by the amplification and sequencing of *VP1*. EVs are associated with various clinical diseases, such as hand, foot, and mouth disease (HFMD), herpangina, respiratory illness, aseptic meningitis, encephalitis, myocarditis, acute flaccid paralysis, acute flaccid myelitis, and sepsis^1^.

Recently, serious infections such as acute amyotrophic myelitis caused by an EV have been reported, raising international concerns about public health risks^2^. Although EV infection routes include fecal, oral, and respiratory; it can also spread through fomites, and vertical infection cases have also been reported^3^. The major countries where EV infections are reported are those in the Western Pacific region, including South Korea, China, Japan, and Taiwan^4^.

The first case of death due to HFMD and nervous system complications was reported in May 2009, and it was officially designated as an infectious disease in June 2009. Since January 2020, in Korea, infectious diseases have been divided into four classes to facilitate responses to the emergence of novel infectious diseases and shifts in epidemic occurrence patterns. Regarding the timing of notification of infectious diseases, the classification criteria stipulate that class 1 infectious diseases must be reported immediately, class 2 and class 3 infectious diseases must be reported within 24 h, and class 4 infectious diseases require sample surveillance to investigate epidemics like Influenza, HFMD, and EV infections among others.

The Korea EV Surveillance System (KESS) had been set up in South Korea in 2006. The KESS is a cooperative network in which national health and environment research institutes along with the regional medical institutions participate, where the number of cooperative medical institutions changes annually depending on their performance and intentions. Hospitals participating in this surveillance collect samples such as feces and throat swabs (14 days before the onset of symptoms) when a patient is suspected of having an EV infection, including HFMD, and transfer the samples to the Institute of Health and Environment Research once a week through a transportation agency. Later, the test results of these samples are sent to the applicable medical institution. The inclusion criteria for the EV surveillance project and the test methods applied were consistent throughout the 10-year period. The KESS involves sentinel surveillance of all suspected EV patients, including herpes stomatitis and HFMD patients, and is not restricted to patients with severe symptoms. Many countries, including South Korea, are currently focused on prevalence and surveillance studies to detect EVs. As a result, changes in serotype distribution and diversity based on the country, year, sporadicity, or nature of the outbreak have been reported.

**Figure 1:**
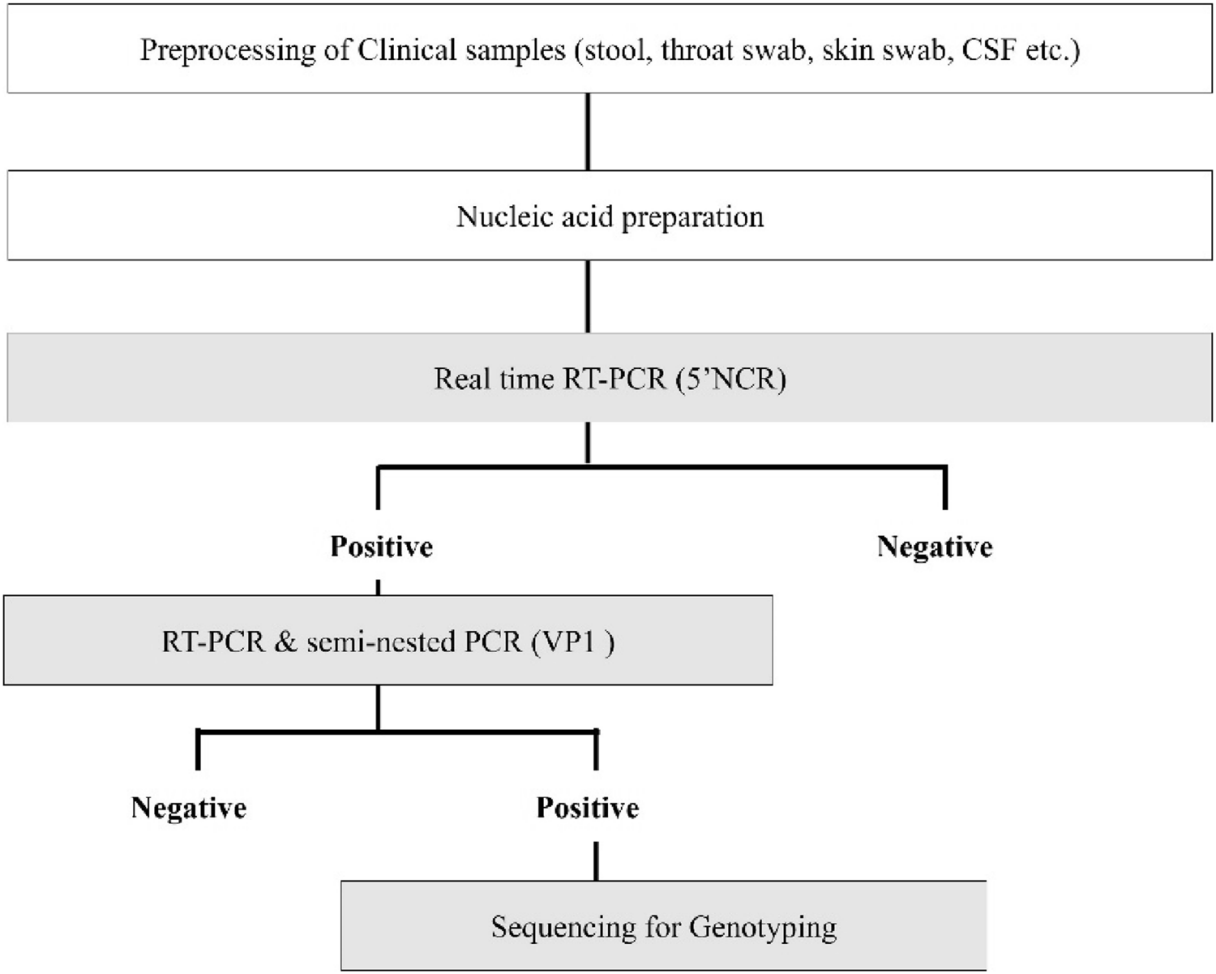
Schematic diagram of detection and genotype investigation of enterovirus (EV) from various clinical samples.

To develop effective preventive measures for EV infection, including vaccines, monitoring the circulating EVs is important. Therefore, in this study, we aimed to evaluate the long-term changes in the prevalence patterns, seasonality, and genotype distribution of EVs in South Korea during 2011–2020, using the genetic testing strategy displayed in Fig. 1.

## Results

### Annual prevalence of EV infectious disease-causing pathogens

**Figure 2:**
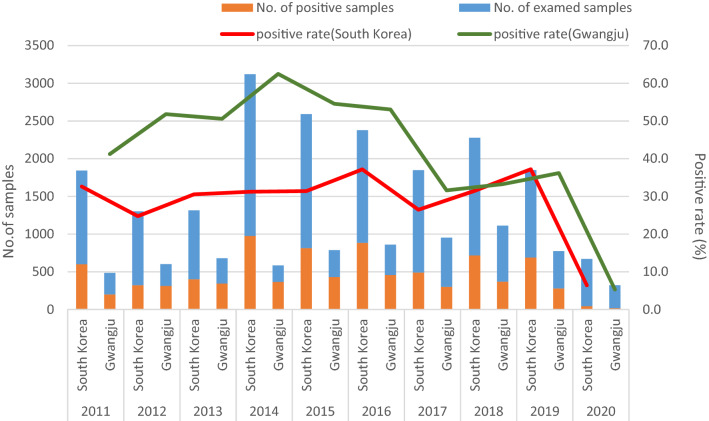
Comparison of EV surveillance data in South Korea and Gwangju.

The overall EV-positive rate was 43.0% (3076/7160 patients). The proportion of EV-positive samples per year ranged from 5.3% (17/321) in 2020 to 62.5% (365/584) in 2014 over the 10-year study period (Fig. 2) inGwangju. In 2011, the detection rates were 41.2% (200/485), 51.8% (312/602) in 2012, and 62.5% in 2014, which gradually increased every year and then decreased to 54.6% (430/788) in 2015, 31.6% (301/952) in 2017, and 36.2% (280/774) in 2019. In 2020, during the coronavirus disease 2019 (COVID-19) pandemic, the detection rate was 5.3%; a decrease of over 90% was observed as compared to that in 2014.

Considering the KESS monitoring data for the same period, of the 19,197 specimens collected, 5935 (30.5%) were laboratory-confirmed as EV-positive. The EV detection rate varied annually, ranging from 6.4% (43/671) in 2020 to 37.2% (884/2379 and 688/1849) in 2016 and 2019 (Fig. 2).

**Figure 3:**
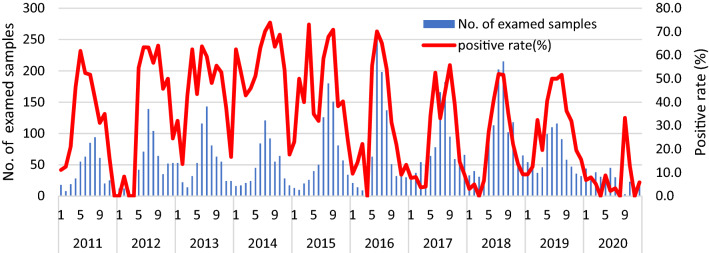
Seasonal pattern of EV circulation in Gwangju, 2011–2020. Bars indicate suspected samples and red curves indicate the positive rate of EV infections per month.

EVs were surveilled yearly, and they appeared to be prevalent mainly between May (spring) to September (early autumn). Figure 3 shows the monthly distribution of the number of suspected EV specimens and the detection rate for 10 years. The positive rate of clinical manifestations was 35.7% (2037/5705) for aseptic meningitis and encephalitis, 73.4% (857/1168) for HFMD, and 63.4% (182/287) for herpangina.

Regarding EV prevalence by clinical manifestations, except during the 2020-COVID-19 pandemic, the annual positive rate for aseptic meningitis ranged between 24.7 and 54.5%, and that for HFMD was in the range of 58.7–93.1%. As it was difficult to distinguish HFMD from herpangina via clinical evaluations, the detection rate was between 20.0 and 71.8% (Table 1).

**Table 1:** Annual EV positive rate from clinical manifestations in Gwangju, South Korea, over 10 years.

Clinical symptom	Total		Aseptic meningitis		HFMD		Herpangina	
Year	No. of test samples (n)	Positive rate (%)	No. of test samples (n)	Positive rate (%)	No. of test samples (n)	Positive rate (%)	No. of test samples (n)	Positive rate (%)
2011	485	41.2	413	32.2	72	93.1	0	0.0
2012	602	51.8	484	43.4	118	86.4	0	0.0
2013	680	50.6	490	39.0	149	83.9	41	68.3
2014	584	62.5	411	54.5	150	83.3	23	69.6
2015	788	54.6	605	51.7	133	67.7	50	54.0
2016	861	53.1	709	51.5	119	60.5	33	60.6
2017	952	31.6	793	24.7	96	68.8	63	61.9
2018	1113	33.2	921	26.9	121	58.7	71	71.8
2019	774	36.2	598	24.7	171	76.6	5	20.0
2020	321	5.3	281	3.9	39	20.5	1	0.0
Total	7160	43.0	5705	35.7	1168	73.4	287	63.4

### Distribution of EV infection by sex and age

Sex and age data were scrutinized using the data collected from 2014 to 2020. Analysis of the male–female ratio among the 5,294 cases (male, 2929; female, 2365) of EV specimens with available sex information showed that the EV infection incidence was higher in males (41.5%, n = 1215) than in females (40.8%, n = 964). However, no significant difference was observed in the positive rate of EV infection between males and females (P > 0.5).

Table 2 shows the age distribution of the 2200 patients with positive test results. Analysis of age groups revealed that the proportion of EV-positive specimens was the highest in 1–4-year-old patients (56.6%, 1257/2200), followed by 5–9-year-old patients (19.8%, 440/2200), < 1-year-old patients, (17.7%, 392/2200), and 10–14-year-old patients (3.5%, 77/2200) (Table 2).

**Table 2:** Age distribution of patients with EV infection in Gwangju, South Korea, between 2014 and 2020.

Age	Number of positive/total patients in the age group						Total
	< 1 years	1–4 years	5–9 years	10–14 years	≥ 15 years	Unknown	
2014	60/100	208/305	66/121	8/25	17/25	6/8	365/584
2015	70/152	237/390	98/192	16/36	2/9	7/9	430/788
2016	86/169	233/336	108/281	22/53	1/8	7/14	457/861
2017	72/201	174/483	43/172	8/72	1/16	3/8	301/952
2018	67/229	226/549	59/223	13/81	1/24	4/7	370/1113
2019	30/160	173/374	63/167	9/50	0/11	5/12	280/774
2020	7/116	6/75	3/76	1/44	0/9	0/1	17/321
Total (%^a^)	392 (17.7)	1257 (56.6)	440 (19.8)	77 (3.5)	22 (1.0)	32 (1.4)	2220/5393

^a^Proportion of EV-positive samples from 2014 to 2020.

**Figure 4:**
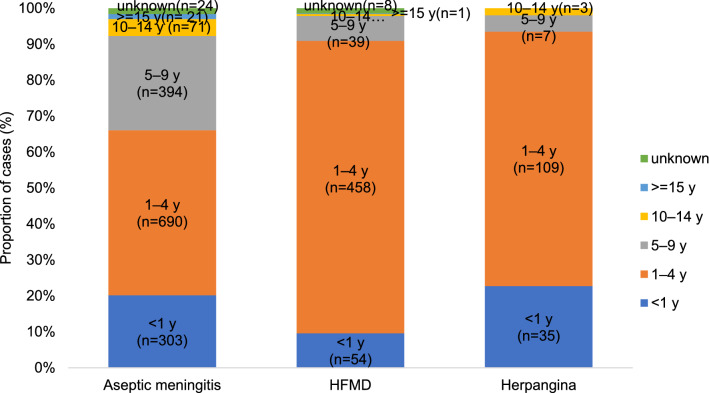
Proportion of EV detections by age group according to clinical manifestations between 2011 to 2020 in Gwangju, South Korea. In the case of hand, foot, and mouth disease (HFMD) and herpangina, 81.3% and 70.8% of the major age groups were between 1 and4 years of age, respectively. Similarly, for aseptic meningitis, 45.9% were between 1 and 4 years, 26.2% between 5 and 9 years, and 20.2% less than 1 year. The infection was uniform in children under 9 years of age.

Age distribution according to the clinical manifestations was similar to that of the overall benign patterns. For HFMD and herpangina, it was 81.3% and 70.8% for 1–4-year-old patients, respectively, but for aseptic meningitis, it was 45.9% for the same age group. Consequently, the rest of the patients grouped as either < 1- or 5–9-year-old patients showed a 20.2% and 26.2% distribution, respectively (Fig. 4).

### Genotype characterization

Table 3 shows the results of EV typing based on partial *VP1* sequencing. Among the EV-positive samples, 70.7% (2174/3076) were successfully genotyped. We identified a total of 35 different EV types belonging to four diverse species and rhinoviruses. EV-A and EV-B were detected in similar proportions, but among these, the most frequently detected EV species was EV-B, with 1119 viruses distributed among 20 genotypes. A total of 1022 viruses belonged to 13 different genotypes within EV-A. One type belonged to EV- C and EV- D each (Table 3).

**Table 3:** Frequencies and number of years identified for individual EV genotypes in Gwangju, South Korea, 2011–2020.

EV species	Type	2011	2012	2013	2014	2015	2016	2017	2018	2019	2020	Total
Enterovirus A (EV-A)	CVA2	6	1	4	9	0	10	12	2	8	0	52
	CVA3	0	0	1	0	0	0	0	0	0	0	1
	CVA4	0	15	1	3	5	9	3	20	2	0	58
	CVA5	0	1	11	1	10	1	**47**	0	2	0	73
	CVA6	0	42	27	41	1	15	6	2	43	**3**	180
	CVA7	0	0	1	0	0	0	0	0	0	0	1
	CVA8	0	9	8	0	0	1	0	2	0	0	20
	CVA10	8	13	4	3	23	3	19	**86**	5	0	164
	CVA12	20	0	0	0	0	0	0	0	0	0	20
	CVA14	1	0	0	0	1	0	0	0	0	0	2
	CVA16	**44**	1	6	**78**	1	18	2	6	8	1	165
	EV-A71	2	50	**73**	15	34	3	6	13	**86**	2	284
	EV-A76	0	2	0	0	0	0	0	0	0	0	2
Enterovirus B (EV-B)	CVA9	1	0	33	2	1	13	1	0	6	**3**	60
	CVB1	0	0	13	14	0	0	0	0	0	0	27
	CVB2	27	0	3	8	0	0	28	0	0	0	66
	CVB3	3	11	0	0	5	6	1	4	1	0	31
	CVB4	5	0	21	0	0	0	44	0	0	0	70
	CVB5	29	0	0	**73**	0	30	2	24	8	0	166
	E3	0	0	18	0	0	0	1	20	0	0	39
	E5	0	0	0	0	0	0	0	1	0	0	1
	E6	0	11	12	0	**47**	1	0	12	0	0	83
	E7	0	7	0	0	0	0	0	0	1	0	8
	E9	21	0	0	17	5	3	0	3	21	0	70
	E11	0	0	0	5	**45**	0	0	58	1	0	109
	E13	0	0	0	0	0	0	1	1	0	0	2
	E14	0	0	5	0	0	0	0	0	0	0	5
	E16	0	0	0	0	14	0	0	0	0	0	14
	E18	8	0	0	0	0	**156**	0	0	0	0	164
	E20	0	0	0	10	0	0	0	0	0	0	10
	E21	0	0	0	0	0	0	1	0	11	0	12
	E25	0	0	0	20	0	0	0	9	0	0	29
	E30	0	**95**	4	0	0	1	15	21	16	1	153
Enterovirus C (EV-C)	CVA19	0	0	0	0	0	0	0	1	0	0	1
Enterovirus D (EV-D)	EV-D68	0	0	0	2	0	0	0	0	0	0	2
Others	Rhino	0	2	1	17	0	0	0	5	3	2	30
Total		175	260	246	318	192	270	189	290	222	12	2174

The most frequently detected EV type in the year are given in bold.

*CV, Coxsackievirus; EV, Enterovirus; E, Echovirus.

**Figure 5:**
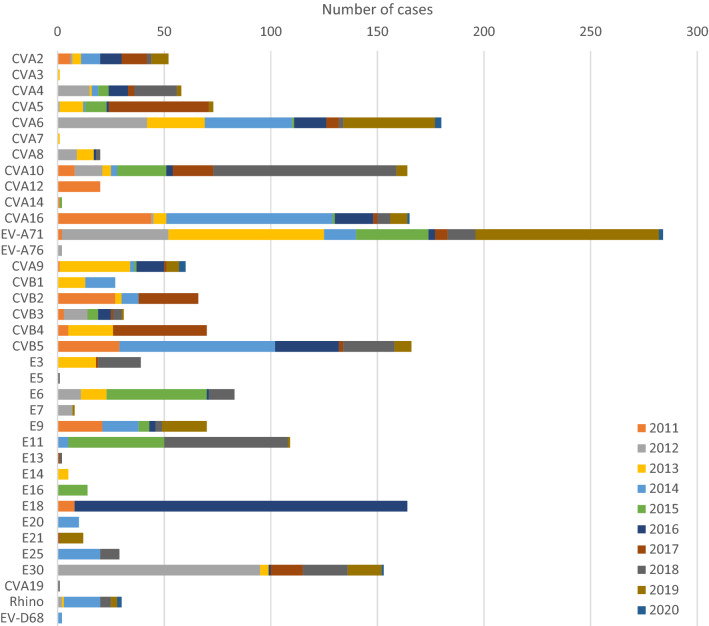
Cumulative number of EV genotypes identified during the period of study (2011–2020) in Gwangju, South Korea. The eight frequently detected EV genotypes were EV-A71 with 13.1% (N = 284), followed by CVA6 (N = 180, 8.3%) and CVB5 (N = 166, 7.6%), followed by CVA16 (N = 165, 7.6%), CVA10 (N = 164, 7.5%), E18 (N = 164, 7.5%), E30 (N = 153, 7.0%), and E11 (N = 109, 5.0%).

**Figure 6:**
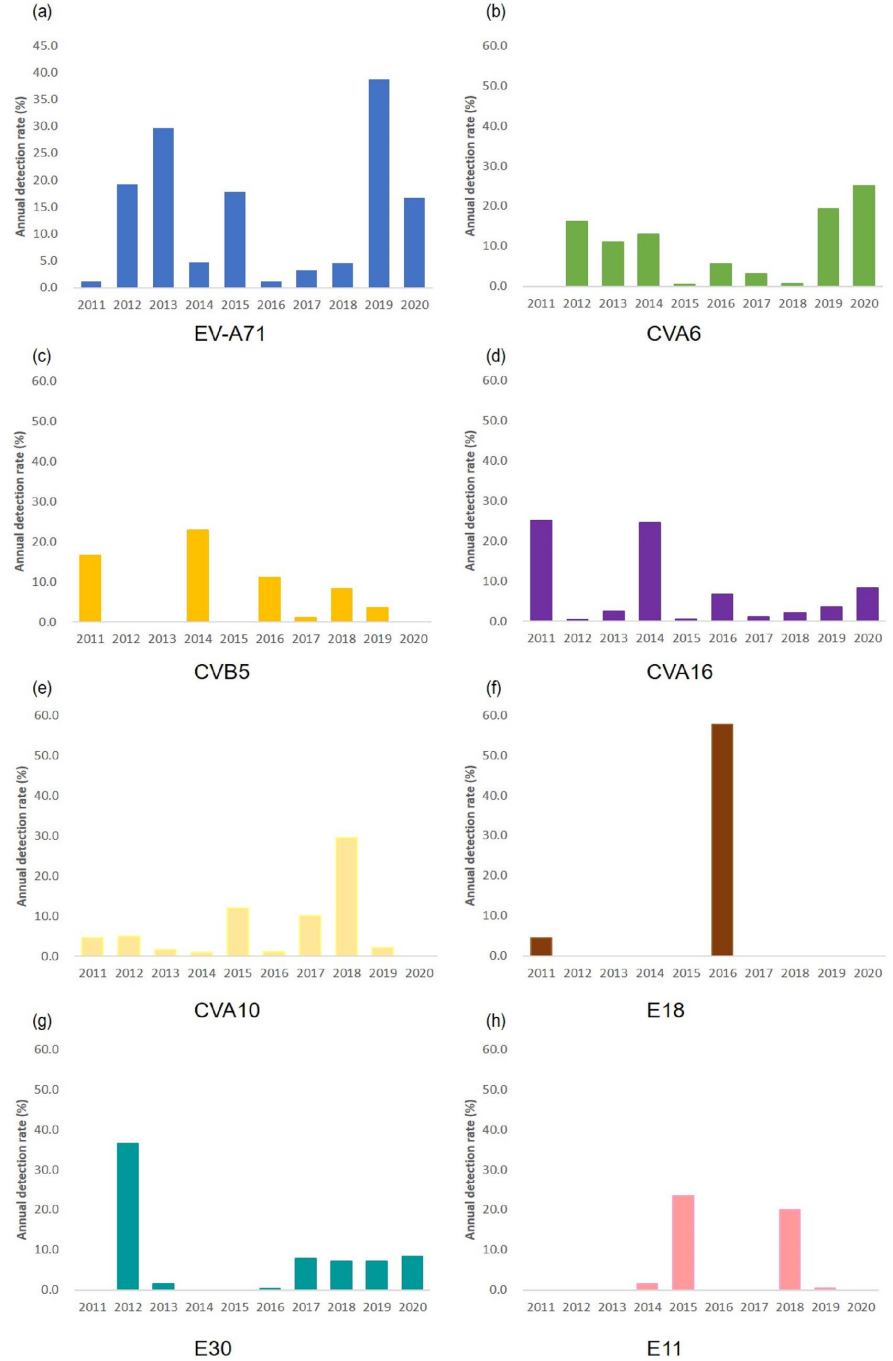
The major EV genotype prevalence patterns showed different characteristics every year.

The eight most frequently observed EVs were EV-A71, accounting for 13.1% of the cases (N = 284), followed by CVA6 (N = 180, 8.3%), CVB5 (N = 166, 7.6%), CVA16 (N = 165, 7.6%), CVA10 (N = 164, 7.5%), E18 (N = 164, 7.5%), E30 (N = 153, 7.0%), and E11 (N = 109, 5.0%), which accounted for over 63% of all EVs detected (Fig. 5). However, the serotype distribution changed frequently with the year; EV-A71, CVA6, CVA16, and CVA10 showed continuous oscillatory detection profile, and CVB5 was detected at a relatively high rate every 1–2 years. E18 and E11 were prevalent in specific years, and E30 caused a major epidemic in 2012 and has been consistently detected at 8% since 2017 (Fig. 6).

### EV genotype by clinical manifestation

**Figure 7:**
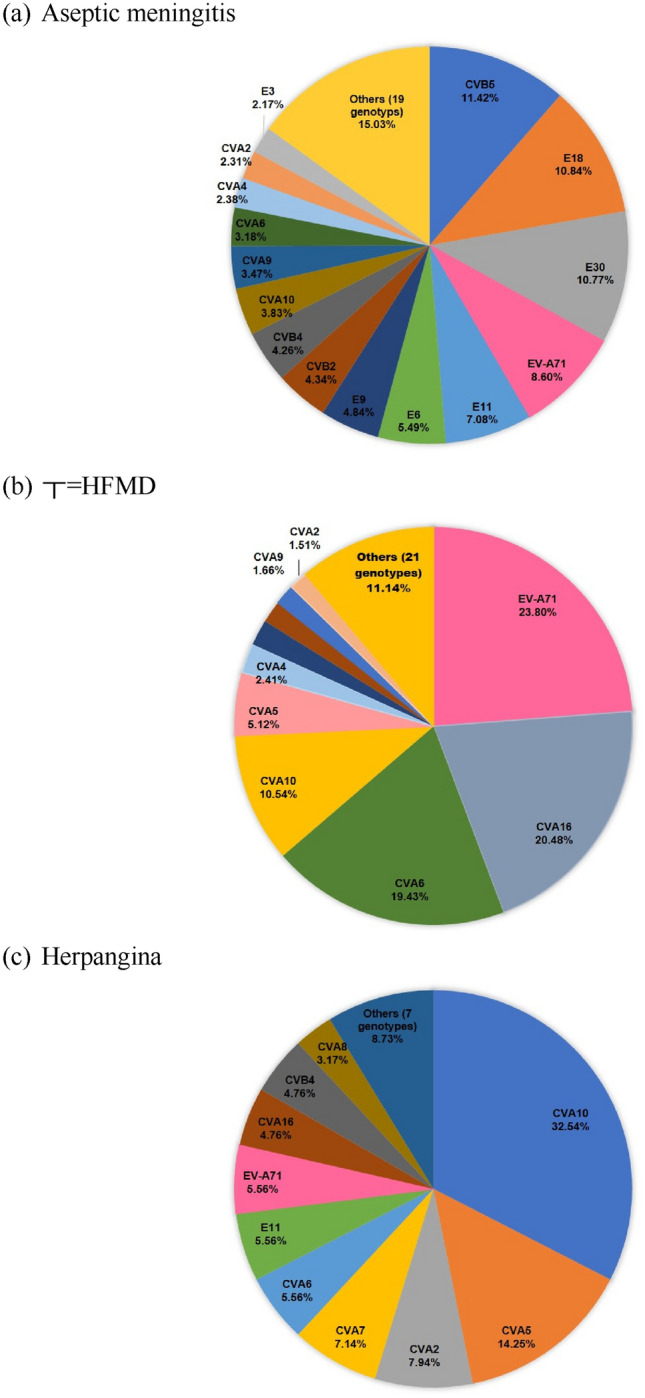
EV genotype distribution of clinical manifestations. Graphic shows the percentage of each genotype (except for untypable cases) of (**a**) aseptic meningitis; (**b**) hand, foot, and mouth disease; (**c**) herpangina (a) Aseptic meningitis showed a similar distribution of CVB5 (N = 158, 11.4%), E18 (N = 150, 10.9%), and E30 (N = 149, 10.8%) belonging to the EV species B group, whereas EV-A71 (N = 158, 23.8%) and CVA16 (N = 136, 20.5%) belonging to EV species A group of (**b**) HFMD. Like HFMD, (**c**) herpangina was dominated by EV species A group, CVA10 (N = 41, 32.5%) and CVA5 (N = 18, 14.3%).

Based on the clinical symptoms or presumptive diagnosis, aseptic meningitis, HFMD, and herpetic stomatitis were classified. Aseptic meningitis was frequently associated with CVB5 (11.42%; 158/1384), E18 (10.84%; 150/1384), and E30 (10.77%; 149/1384). Among HFMD cases, EV-A71 infection was identified in 23.80% (158/664), CVA16 in 20.48% (136/664), and CVA6 in 19.43% (129/664) of the samples. In patients with signs of herpangina, CVA10 was the dominant genotype in 22.53% (41/182), CVA5 in 9.89% (18/182), and CVA2 in 5.49% (10/182) of the cases (Fig. 7).

## Discussion

EV infection is a major pediatric disease in Asian countries and causes large outbreaks, with a substantial number of cases leading to serious and at times fatal complications^5^. Globally, the same type of outbreak has not yet been identified, but the clinical characteristics of this disease and the serotype epidemiology have changed over the last 10 years^6–13^.

To understand EV epidemiology, herein, we presented longitudinal data reflecting changing EV prevalence patterns over 10 years in South Korea. As 37.3% of the KESS data obtained during the monitoring period were from the Gwangju region, it can be observed that these data reflected the results of EV surveillance in South Korea.

The detection rate increased yearly, peaking at 62.5% in 2014 and then gradually decreasing to approximately 30% in 2019. Particularly, low sample numbers (n = 321) and low positive rates (5.3%) were observed in 2020. This may be owing to the intensified hygiene regulations, use of masks in public, social distancing, and a complete shutdown of schools and daycares implemented because of the COVID-19 pandemic^14^.

In our study, although the data by age and sex were limited from 2014 to 2020, the highest sample numbers and positive rates of EV infection were observed in the 1–4-year-old group, regardless of the EV genotype and clinical symptoms. This is consistent with the previous results indicating that pediatric patients are more susceptible to EV infection, although most of the EV-suspected patients were under the age of 15 years (98.9%, n = 5334). Additionally, this age group that showed EV infection susceptibility also reflected higher positive rates of HFMD and herpangina than aseptic meningitis. These results are also consistent with those of a previous study in which most EV cases involved children aged between 2 and 5 years in South Korea from 1999 to 2011^15^.

Several studies have observed that males are more susceptible to infections than females, which was due to the difference in immunity between them^16^. However, we did not observe any significant differences in the positive rates of EV infection between male and female patients (OR: 1.03).

During the study period, genotyping was achieved through *VP1* amplification in 2174 (70.7%) of the 3076 enteroviral infections. Regarding EV spectrum diversity, on average, approximately 11 different genotypes were isolated in a given year (range: 8–14). From 2011 to 2020, 35 EV genotypes were prevalent in South Korea, and the predominant genotype changed every year. The eight commonly detected EV types were EV-A71 (13.1%), CVA6 (8.3%), CVB5 (7.6%), CVA16 (7.6%), CVA10 (7.5%), E18 (7.5%), E30 (7.0%), and E11 (5.0%). Similarly, in a study from 2012 to 2019 in Korea, the most frequently detected type was EV-A71 (15.8%), followed by E30 (10.0%), CVB5 (7.8%), CVA6 (7.3%), and CVA10 (7.4%)^17^. The most frequently detected genotype was EV-A71, but the species was EV-B. The differences in the detection rates of EV types are due to the differences in the detection of genotypes according to clinical manifestations. EV-A71, CVA6, CVA10, and CVA16, which constitute EV-A, were detected at the highest rate and were associated with HFMD and herpangina; whereas, EV-B, CVB5, E18, and E30 were mainly associated with aseptic meningitis. This is consistent with previous studies that suggest EV-A is highly prevalent but is not the predominant species^18^, and HFMD and herpangina outbreaks in Asia occur due to EV-A71, CVA6, and CVA16^19,20^.

Most EV studies to date have only reported total EV genotypes that do not discriminate diseases by type or have been limited to reporting EV genotypes for type-specific diseases (such as HFMD and herpangina). However, our results are more precise because we analyzed EV types based on clinical symptoms and categorized the different EV types in total EV infections over a decade.

EV-A71 is the most important causative agent of HFMD and herpangina-associated neurological and cardiac complications, which may lead to HFMD outbreaks associated with high mortality rates in children^21^. The first reported EV71 infection in South Korea occurred between 1989 and 1990^22^. Since 2008, outbreaks of EV-A71 infection associated with neurological involvement (HFMD with central nervous system complications) have been reported^23,24^. Recently, EV-A71 molecular typing has been routinely conducted for the early identification of emerging strains in numerous countries worldwide, and the major sub-genotypes have been reported in such countries each year^17,24–27^. However, in this study, EV-A71 molecular typing was not performed, which may be done in the near future to identify the viral characteristics by clinical symptoms, age, and period.

CVA6, the second most frequently identified pathogen in this study, has long been known as one of the major pathogens causing HFMD in Asia that has replaced EV-A71 and CVA16 in recent years^28–30^. In South Korea, during the decade investigated, CVA6 had the third-highest detection rate from 2012 to 2013 and the second-highest detection rate in 2019. These results are similar to the KESS surveillance results obtained from 2012 to 2019^17^.

CVB5 is a commonly reported EV type in the United States, China, and Europe, and is associated with acute viral meningitis^31–34^. It was the second most frequent genotype detected in 2011, 2014, and 2015, and was detected intermittently before 2016 but appeared every year after that (except in 2020).

The next most comparably detected genotypes were CVA16, CVA10, and E18. Among them, CVAs were consistently detected every year in suspected HFMD and herpangina cases, but E18 appeared only in 2011 and 2016 during the study period. E18 is an important pathogen in aseptic meningitis^35^ and has been associated with its outbreaks^36,37^. E18 was first identified in 1955 in the U.S. In 2016, it was confirmed to be the causative agent of the meningitis outbreak.

EV-D68 was reported only as a cause of sporadic outbreaks until 2005. In recent years, many reports from different countries have described an increasing number of patients with respiratory diseases due to EV-D68 infection. In addition, EV-D68 has become one of the major EV genotypes associated with acute flaccid paralysis and cranial nerve dysfunction in children^38^. However, in the present study, EV-D68 was confirmed in only two cases in 2014, and the associated clinical symptoms were aseptic meningitis in one case and HFMD in the other. This is consistent with the fact that there have been no reports of the EV-D68 group outbreak in South Korea. However, it is necessary to compare and analyze it with the subgenotype that has been reported to cause acute flaccid paralysis, by whole-length genomic analysis which could offer further critical insights.

The present study had some limitations. First, most of the target monitoring hospitals that provided specimens are concentrated in second and third tertiary pediatric hospitals. Second, *VP1* polymerase chain reaction (PCR), which was performed for genotyping, was successful in only approximately 71% (2174/3076) of all EV-positive samples. Finally, subtyping of major EV serotypes was not performed. Nevertheless, our findings provide information on the 10-year EV epidemiology in Gwangju, South Korea, using the KESS. This surveillance provides valuable data on epidemiological patterns and clinical manifestations associated with specific genotypes.

As the continuous laboratory monitoring system of EVs indicate various viruses with different clinical symptoms, it is crucial to identify the viral subtypes and prevent diseases. Furthermore, there are no antiviral treatments that are specific to EV infections. These emerging strains should be the primary targets for the preparation of EV vaccines.

Therefore, our results will provide a scientific basis for basic data on vaccine development and therapeutic research as well as response to infectious diseases.

## Methods

### Ethics approval and consent to participate

The study was approved by the Institutional Review Board of the Korea Centers for Disease Control and Prevention (approval number 2018-08-02-2C-A). All experiments were performed in accordance with relevant guidelines and regulations. In addition, we have obtained the informed consent of all subjects and/or legal guardians by stating that "You cannot claim your rights to the development of new drugs or diagnostic tools, etc., or the application for patents, etc., based on the results of research using your human derivatives, etc., and that research using human derivatives provided by you will be published in the name of the researcher and your personal information will not be revealed".

### Clinical sample collection

A total of 7160 specimens (stool, throat swab, skin swab, and cerebrospinal fluid [CSF]) were collected from patients suspected of EV infections in ten local hospitals between 2011 and 2020 in Gwangju, Korea. The data covers the southeast region of Korea, as major tertiary care centers and primary and secondary hospitals are in Gwangju. From 2014 to 2020, when the age and sex data of patients were available, the median age of the study population was 2 years, where the patients ranged from 1 month to 81 years of age, with a male-to-female ratio of 1.24.

Stool samples were diluted with phosphate-buffered saline by approximately 10%, shaken vigorously for 15 min in a mechanical shaker, and centrifuged at 13,000 g for 10 min at 4 °C, and the supernatant was used. Other samples (throat swabs, skin swabs, and CSF) were used directly without any pre-treatment.

### RNA extraction and real-time reverse transcription polymerase chain reaction for EV detection

Viral RNA was extracted using the QIAamp Viral RNA Mini Kit (Qiagen, Hilden, Germany) according to the manufacturer’s instructions. The RNA was suspended in an elution buffer in a final volume of 60 μL and stored at − 80 °C until use.

To detect EVs, real-time reverse transcription-PCR (RT-PCR) using EV-specific probes and primers reacting to the highly conserved 5ʹ non-coding region (5’NCR) was performed^4^.

### EV genotyping and phylogenetic analysis

For genotype identification, the capsid protein VP1 region was amplified using RT-PCR and a semi-nested PCR kit (iNtRON Biotechnology, Gyeonggi, Korea). All RT-PCR products were sequenced. Forward and reverse sequences were assembled using ClustalW and queried against sequences in GenBank using BLAST and the Enterovirus Genotyping Tool Version 1.0^38^. Figure 1 shows the schematic diagram of EV genetic testing.

### Approval for human experiments

The study was approved by the Institutional Review Board of the Korea Centers for Disease Control and Prevention (approval number 2018-08-02-2C-A).

## Data Availability

The datasets generated during and/or analyzed in the current study are available from the corresponding author upon reasonable request.
